# Chromosome phylogeny of the subfamily Pitheciinae (Platyrrhini, Primates) by classic cytogenetics and chromosome painting

**DOI:** 10.1186/1471-2148-10-189

**Published:** 2010-06-20

**Authors:** Liane FM Finotelo, Paulo JS Amaral, Julio C Pieczarka, Edivaldo HC de Oliveira, Alcides Pissinati, Michaela Neusser, Stephan Müller, Cleusa Y Nagamachi

**Affiliations:** 1 Laboratório de Citogenética, Instituto de Ciências Biológicas, Universidade Federal do Pará, Belém, Brazil; 2 Institut für Humangenetik, Klinikum der Ludwig-Maximilians-Universität, Munich, Germany; 3 CNPq Researcher, Belém, Brazil; 4 FAPESPA Doctorship Scholarship in Neurociences and Celular Biology, Belém, Brazil; 5 FAPESPA Doctorship Scholarship in Genetics and Molecular Biology, Belém, Brazil; 6 Centro de Primatologia do Rio de Janeiro, Niterói, Brazil

## Abstract

**Background:**

The New World monkey (Platyrrhini) subfamily Pitheciinae is represented by the genera *Pithecia*, *Chiropotes *and *Cacajao*. In this work we studied the karyotypes of *Pithecia irrorata *(2n = 48) and *Cacajao calvus rubicundus *(2n = 45 in males and 2n = 46 in females) by G- and C-banding, NOR staining and chromosome painting using human and *Saguinus oedipus *whole chromosome probes. The karyotypes of both species were compared with each other and with *Chiropotes utahicki *(2n = 54) from the literature.

**Results:**

Our results show that members of the Pitheciinae have conserved several chromosome forms found in the inferred ancestral Platyrrhini karyotype (associations of human homologous segments 3a/21, 5/7a, 2b/16b, 8a/18, 14/15a and 10a/16a). Further, the monophyly of this subfamily is supported by three chromosomal synapomorphies (2a/10b, an acrocentric 15/14 and an acrocentric human 19 homolog). In addition, each species presents several autapomorphies. From this data set we established a chromosomal phylogeny of Pitheciinae, resulting in a single most parsimonious tree.

**Conclusions:**

In our chromosomal phylogeny, the genus *Pithecia *occurred in a more basal position close to the inferred ancestor of Platyrrhini, while *C. c. rubicundus *and *C. utahicki *are closely related and are linked by exclusive synapomorphies.

## Background

The subfamily Pitheciinae includes the genera *Pithecia *Desmarest, 1804, *Chiropotes *and *Cacajao *Lesson, 1840. *Pithecia *comprises five species: *Pithecia pithecia*, *Pithecia monachus*, *Pithecia irrorata *(each two subspecies), *Pithecia albicans *and *Pithecia aequatorialis *(both monotypic) [1]. *Chiropotes *is represented by the two species *Chiropotes albinasus *(monotypic) and *Chiropotes satanas *(three subspecies) [2], and *Cacajao *by *Cacajao calvus *(four subspecies) and *Cacajao melanocephalus *(two subspecies) [3]. The geographic distribution of this subfamily is restricted to the Amazon region. Recent morphological, karyotypic and molecular data pointed to a new species for *Chiropotes, C. israelita*, and indicated that the subspecies *Chiropotes satanas utahicki *should be accepted as a full species [4]. The majority of the Pitheciinae species are listed as endangered (http://www.cites.org). 

It is accepted that the Pitheciinae represent a monophyletic clade [4-7]. Several studies using morphological [5-7], molecular [8,9] and cytogenetic [10] traits suggest that *Chiropotes *and *Cacajao *are sister taxa and place *Pithecia *most basal within this clade. It was further suggested that the Pitheciinae are a sister group of *Aotus *and *Callicebus *[5], while more recent molecular phylogenetic analyses supported an association of Pitheciinae and *Callicebus*, but associate *Aotus *with Callitrichidae [8,11-14].

To date, cytogenetic studies of members from this subfamily are still rare. The karyotypes of *C. albinasus, P. monachus, P. aequatorialis *and *P. albicans *have not yet been described so far, while for others, for example for *C. melanocephalus*, only the diploid chromosome number was published [15]. Moura-Pensin *et al*. [10] were the first to present a broader comparative cytogenetic study, including *Pithecia irrorata*, *Chiropotes satanas chiropotes*, *Chiropotes satanas utahicki *and *Cacajao calvus rubicundus*. *Cacajao *shows the lowest diploid chromosome number among Pitheciinae with 2n = 46 in females and 45 in males [10,15-17]. *Pithecia *has 2n = 48 chromosomes [18], and *Chiropotes *has the highest diploid number with 2n = 54 [10,19]. Only *Chiropotes utahicki *and *C. israelita *were so far analyzed by chromosome painting using human whole chromosome probes [19]. Stanyon *et al*. [19] showed that *Chiropotes *retained all human homologous syntenic associations proposed for the ancestral Platyrrhini karyotype, but also include several derived chromosome forms that are exclusive to this genus.

With the aim to establish detailed chromosomal phylogenies of the three genera of Pitheciinae, we studied the karyotypes of the species *Pithecia irrorata *and *Cacajao calvus rubicundus *using both classic cytogenetics and chromosome painting with human and *Saguinus oedipus *whole chromosome probes.

## Methods

Chromosome preparations were obtained from whole blood cultures of one female and two male *Pithecia irrorata *(PIR) individuals kept at the Parque Zoobotânico Gavião Real, Capitão Poço, Para, Brazil, of two females from the Centro Nacional de Primatas, Ananindeua, Para, Brazil, and of a male and a female *Cacajao calvus rubicundus *(CCR) individual kept at the Centro de Primatologia do Rio de Janeiro, Rio de Janeiro, Brazil.

G-banding, using Wright stain [20], C-banding [21] and NOR-staining [22], followed standard procedures. FISH experiments using human whole chromosome paint probes 1-22, X and Y, and *S. oedipus *paint probes (22 autosomes, X and Y) were performed as previously described [23-25]. While both human probes were hybridized for CCR and PIR, only PIR is queried with SOE paints. Paint probes were labeled by DOP-PCR [26] using Biotin-dUTP, Digoxigenin-dUTP (Roche) and TAMRA-dUTP (Biosystems/PE Applied). After classical staining, metaphases were photographed using a Zeiss III microscope with Kodak Imagelink HQ film. FISH/DAPI stained metaphase images were captured with a CCD camera (AxioCam MR monochrome, or Photometrics C250/A, equipped with a KAF1400 chip, respectively) coupled to a Zeiss Axiophot microscope. The images were analyzed using Adobe Photoshop software version CS3 and Corel Photo Paint 10. Chromosomes were identified by computer enhanced DAPI banding (Axiovision 3.0).

For phylogenetic analysis a binary data matrix was established, based on the presence or absence of discrete chromosomal characters obtained by comparative analysis of both chromosome painting patterns derived from human whole chromosome probes and by G-banding patterns in the Pitheciinae species included in our study and from the literature [19]. *Brachyteles arachnoides *[24] and *Cebus apella *[25] were used as outgroups. We attributed "0" (zero) value to the absence of a character (an association or a syntenic group that is conserved or changed by chromosome rearrangements) and "1" (one) value to the presence of the character. These data were subjected to maximum parsimony analysis (PAUP 4.0 software; Phylogenetic Analysis Using Parsimony), using the exhaustive search option. The relative stability of nodes was assessed by bootstrap estimates.

## Results

### Classical cytogenetics

**Figure 1:**
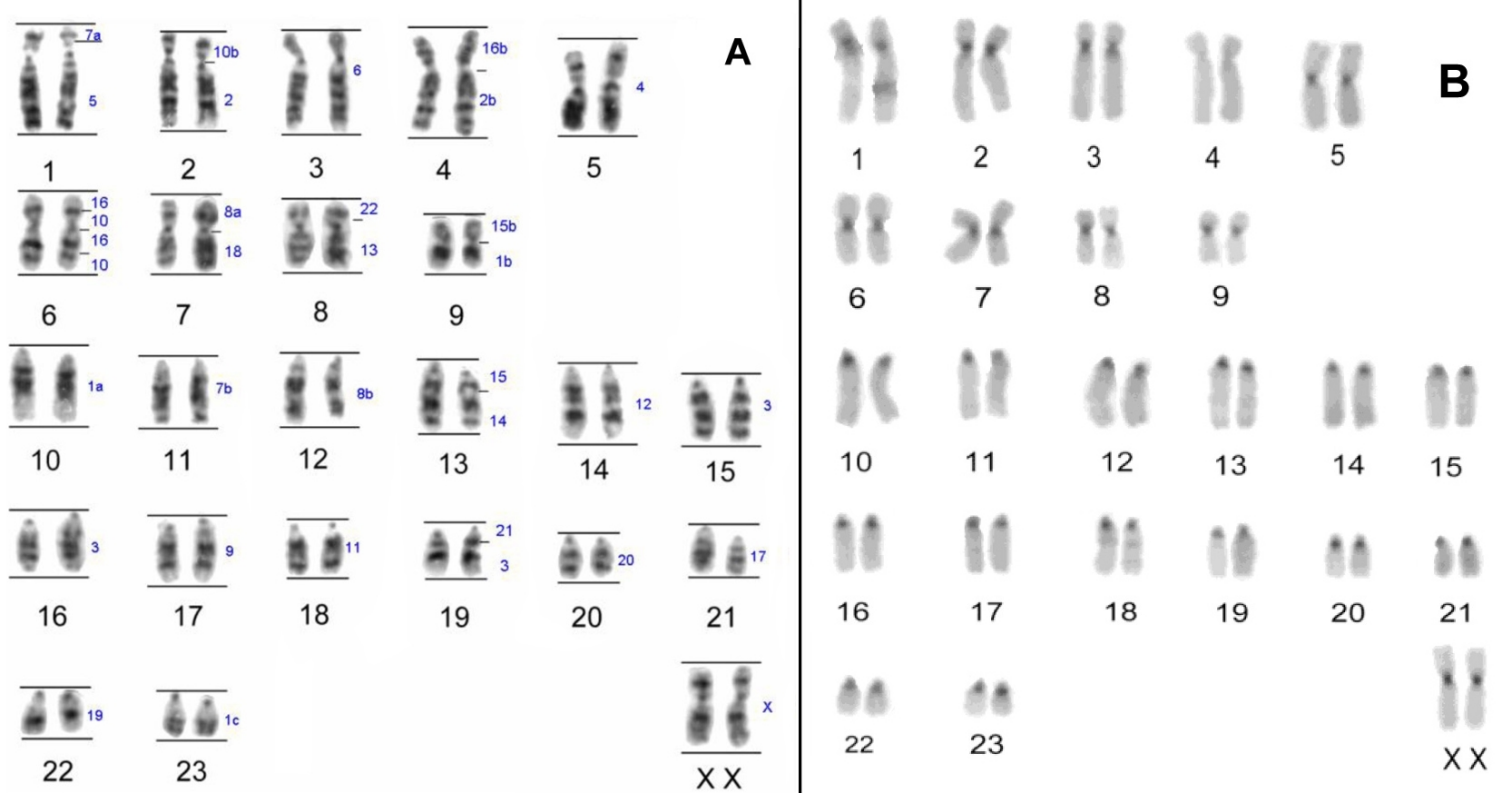
**G- (A) and C-banding (B) in *P. irrorata***.

*P. irrorata *has 2n = 48 chromosomes, with nine metacentric or submetacentric and 14 acrocentric autosome pairs. The X chromosome is a medium sized submetacentric. The G-banded karyotype is shown in the Figure 1A. C-banding highlighted constitutive heterochromatin in the centromeres of all chromosomes and an interstitial band in the long arm of pair 18 (Figure 1B).

**Figure 2:**
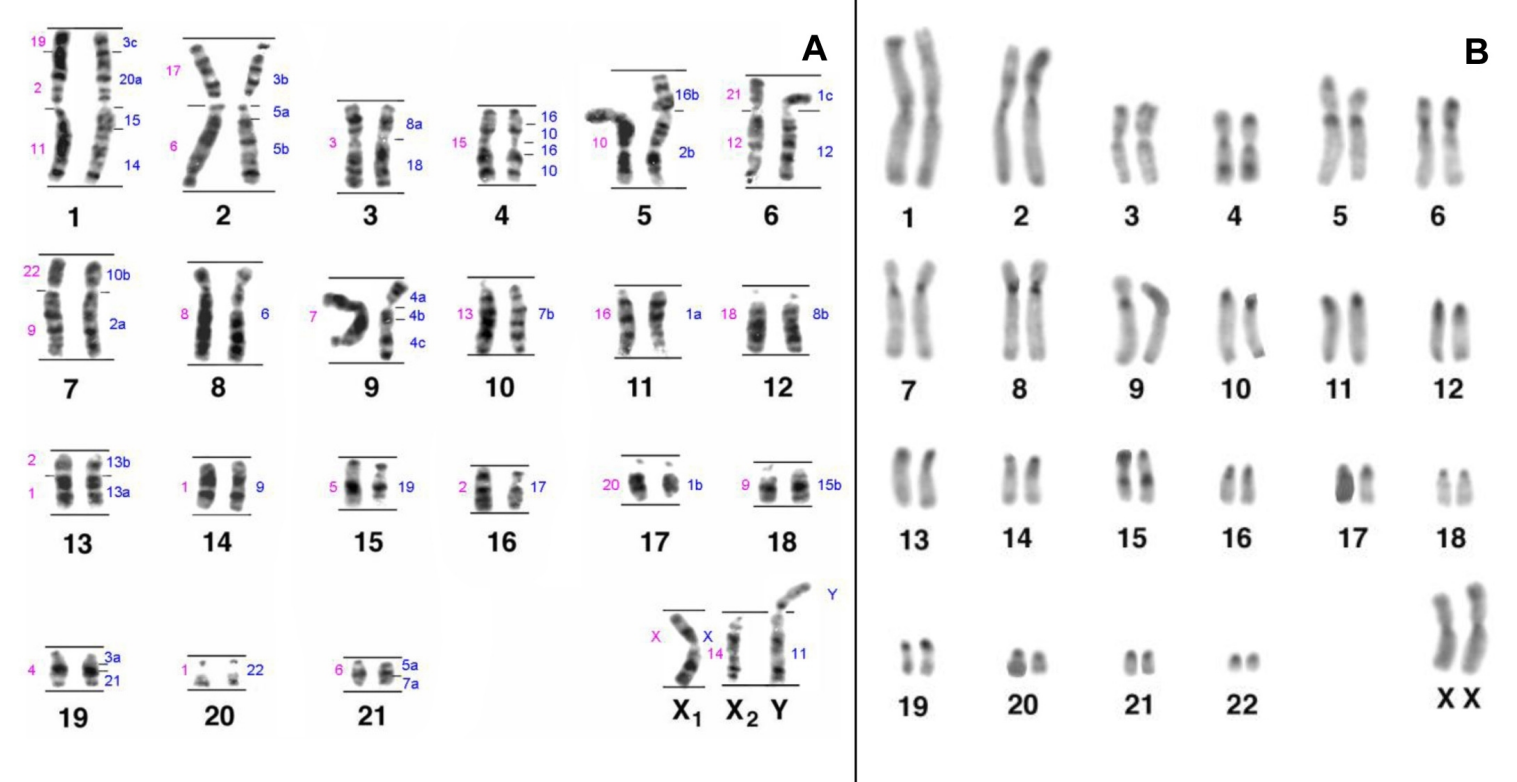
G - (A) and C-banding (B) in *C. c. rubicundus.*

*C. c. rubicundus *has 2n = 45 chromosomes in males and 2n = 46 in females as a result of a Y-autosomal translocation, causing a multiple sex chromosome system X_1_X_2_Y (Figure 2A). The autosomal set is composed by nine meta- or submetacentric and 13 acrocentric pairs. The X_1 _chromosome is a medium sized submetacentric, homologous to the X of other primates. The X_2 _chromosome is a medium sized acrocentric, corresponding to the original homologue of the autosome translocated to the Y chromosome. Constitutive heterochromatin (Figure 2B) was observed in centromeric regions of all autosomes. In addition, secondary C-bands were observed on pairs 1, 2 and 3, in the distal regions of chromosome pairs 4p, 5p, 6p and 4q and proximally on chromosome pair 15q.

**Figure 3:**
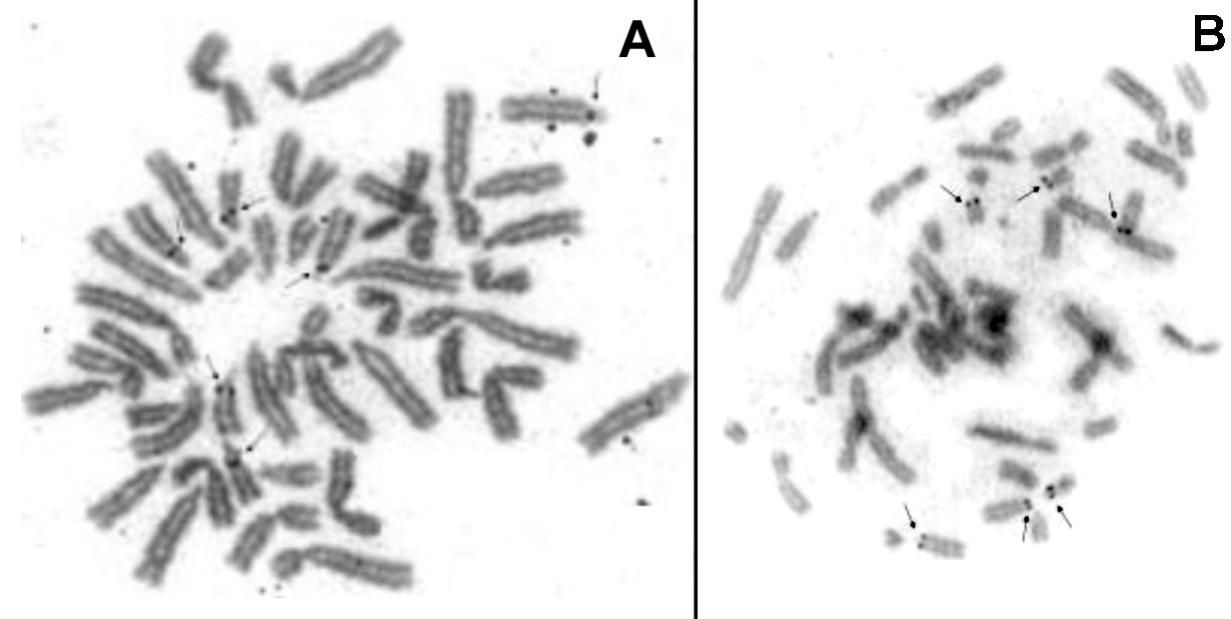
**NOR-staining in *P. irrorata *(A) and *C. c. rubicundus *(B). NOR-regions are highlighted by arrows**.

NOR-staining was found in the proximal region of the long arm of six acrocentric chromosomes both on *P. irrorata *(Figure 3A) and *C. c. rubicundus *(Figure 3B).

### Molecular cytogenetics

#### Pithecia irrorata

**Figure 4:**
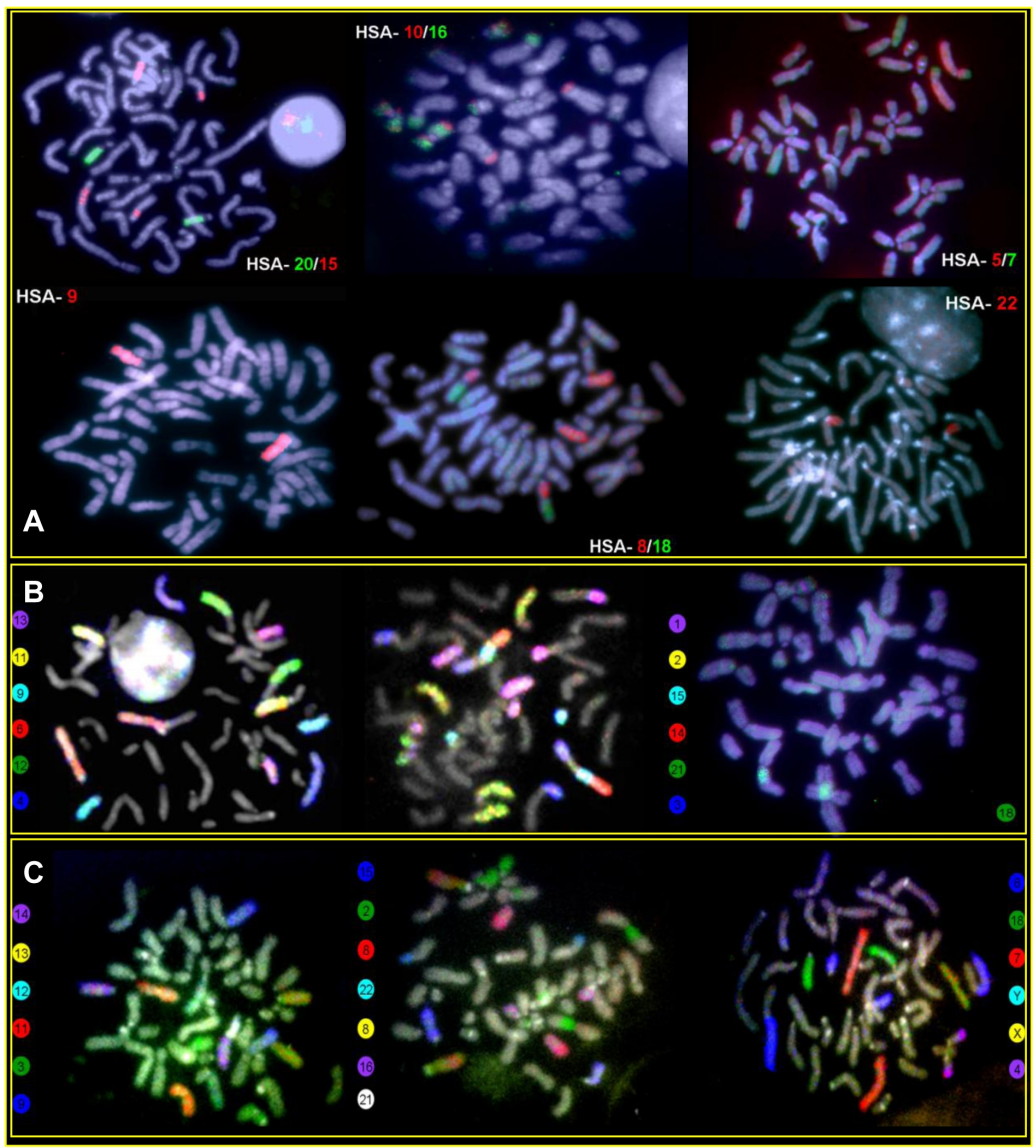
**Representative FISH-images from cross-species chromosome painting experiments using human probes in *P. irrorata *(A), human (B) and *S. oedipus *probes (C) in *C. c. rubicundus***. Beside each metaphase the respective probe composition and color assignment is given

The homology map between human (HSA) and *P. irrorata *was established using chromosome paint probes (Figure 1A). Human probes showed 32 hybridization signals per haploid chromosome set (Figure 4A; Table 1). Fourteen autosomes and the X showed conserved synteny. From these, eight autosomes hybridized an entire *P. irrorata *chromosome (HSA 4, 6, 9, 11, 12, 17, 19, 20) and six were found associated with other chromosomes (HSA 5, 13, 14, 18, 21, 22). The remaining eight human autosome probes produced multiple signals in different chromosomes of *P. irrorata*. HSA 2, 7, 8, 10, 15, and 16 each hybridized two chromosomes, while HSA 1 and 3 each produced signals on three chromosomes per haploid set. Six chromosomes of *P. irrorata *correspond to the human syntenic associations 5/7, 2/16, 10/16, 8/18, 15/14 and 3/21, also previously found in the putative ancestral karyotype of Platyrrhini.

**Table 1:** Left (columns 1-3): Homology between human (HSA), *P. irrorata *(PIR) and *C. c. rubicundus *(CCR) chromosomes, Right (columns 4 and 5): Homology between *S. oedipus *(SOE) and *C. c. rubicundus *(CCR) chromosomes.

HSA	PIR	CCR	SOE	CCR
1	10, 9 (q) and 23	11, 17 and 6 (p)	1	13 (distal q), 14 and 20
2	2 (q) and 4 (q)	5 (q) and 7 (q)	2	1 (proximal p), 13 (proximal q) and 16
3	15, 16 and 19 (distal q)	19 (proximal q), 2 (p) and 1 (distal p)	3	3
4	5	9	4	19
5	1 (proximal q + p)	21 (proximal q) and 2 (q)	5	15
6	3	8	6	2 (q) and 21
7	1 (distal p) and 11	21 (distal q) and 10	7	9
8	7 (p) and 12	3 (p) and 12	8	8
9	17	14	9	7 (q) and 18
10	6 (proximal p- distal q) and 2(p)	4 (proximal p- distal q) and 7(p)	10	5
11	18	Y-autosome	11	1 (q)
12	14	6 (q)	12	6 (q)
13	8 (proximal q + p)	13	13	10
14	13 (distal q)	1 (distal q)	14	Y-autosome
15	9 (p) and 13 (proximal q)	1 (proximal q) and 18	15	4
16	6 (distal p- proximal q) and 4(p)	4 (distal p- proximal q) and 5(p)	16	11
17	21	16	17	2 (p)
18	7 (q)	3 (q)	18	12
19	22	15	19	1 (distal p)
20	20	1 (proximal p)	20	17
21	19 (proximal q)	19 (distal q)	21	6 (p)
22	8 (distal p)	20	22	7 (p)
X	X	X	X	X

#### Cacajao calvus rubicundus

Hybridizations of human paint probes resulted in 33 FISH-signals per haploid set of *C. c. rubicundus *(Figure 4B; Table 1). Thirteen human autosomes and the X showed conserved synteny. From these, HSA 4, 6, 9, 11, 13, 17, 19 and 22 probes hybridized onto a whole chromosome and HSA 12, 14, 18, 20, 21 were found in syntenic association. The remaining nine probes produced multiple signals in different chromosomes of *C. c. rubicundus*. HSA 2, 5, 7, 8, 10, 15, and 16 paints each hybridized two chromosomes, whereas HSA 1 and 3 probes each hybridized three chromosomes. Nine chromosomes of *C. c. rubicundus *presented the syntenic associations 1/12, 2/10, 2/16, 3/20/15/14, 3/5, 3/21, 5/7, 8/18 and 10/16. Human chromosome 11 was identified as the autosome involved in the Y-autosome translocation. Figure 2A summarizes the mapping of human chromosomes to the G-banded karyotype of *C. c. rubicundus*.

Hybridizations of *S. oedipus *(SOE) probes showed 28 signals per haploid chromosome set of *C. c. rubicundus *(Figure 4C; Table 1). SOE autosome 3, 4, 5, 7, 8, 10, 13, 14, 15, 16, 18 and 20 probes each hybridized an entire chromosome and SOE 11, 12, 17, 19, 21 and 22 were found conserved but in association with other chromosomes. The remaining four probes each produced multiple signals in different chromosomes of *C. c. rubicundus*. SOE 6 and 9 hybridized onto two chromosomes and SOE 1 and 2 three chromosomes each per haploid set, respectively. Five chromosomes of *C. c. rubicundus *correspond to the syntenic associations 19/2/11, 17/6, 21/12, 22/9 and 2/1. The Y-autosome translocation involved the SOE 14 homologue. Figure 2A gives an overview of the mapping of the SOE chromosome probes to the G-banded karyotype of *C. c. rubicundus*.

### Phylogenetic analysis

**Figure 5:**
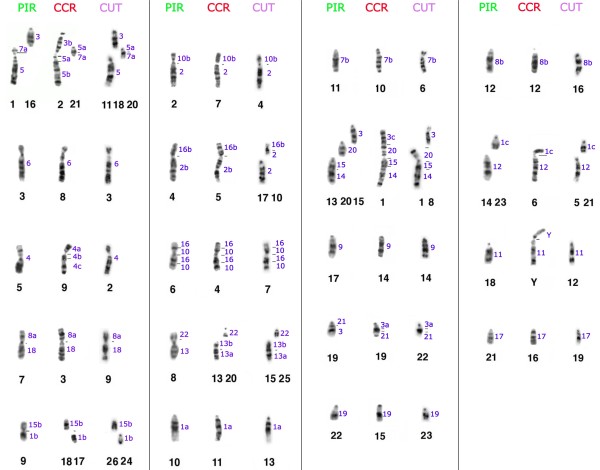
**Comparative karyotype analysis of *P. irrorata, C. c. rubicundus *and *C. utahicki ***[19]**by G-banding and FISH using human whole chromosome probes. **The banded *C. utahicki *chromosomes were obtained from a previously unpublished metaphase [10].

The comparative analysis of the *P. irrorata, C. c. rubicundus *(this study) and *C. utahicki *[19] karyotypes by G-banding and FISH with human whole chromosome probes is summarized in Figure 5. The results showed that the chromosomal differences found among the three taxa are consequence of centric fusions and fissions, pericentric and paracentric inversions, tandem fusions and a Y-autosome translocation (Table 2).

**Table 2:** Derived rearrangements that led to chromosome forms found in the three Pitheciinae taxa, taking into account data from chromosome painting with human whole chromosome probes (HSA) and from G-banding on *P. irrorata *(PIR), *C. c. rubicundus *(CCR) and *C. utahicki *(CUT - [**19**]).

HSA	PIR	CCR	CUT	CHROMOSOME REARRANGEMENTS
10a/16a	6	4	7	Paracentric inversion
7b	11	10	6	Pericentric inversion
11	18	Y	12	Y-autosome translocation
3a/21	19	19	22	Paracentric inversion
5/7a 3b	1 16	2 21	11 18 20	Inversions (pericentric and paracentric); centric fission and *tandem *fusion
2b/16	4	5	10 17	Pericentric inversion; centric fission
13 22	8	13 20	15 25	Pericentric inversion; centric fission
1b 15b	9	17 18	24 26	centric fission
14/15a 20 3c	13 20 15	1	1 8	*Tandem *fusion; centric fission; pericentric inversion
1c 12	23 14	6	21 5	Centric fission and fusion; pericentric inversion

**Figure 6:**
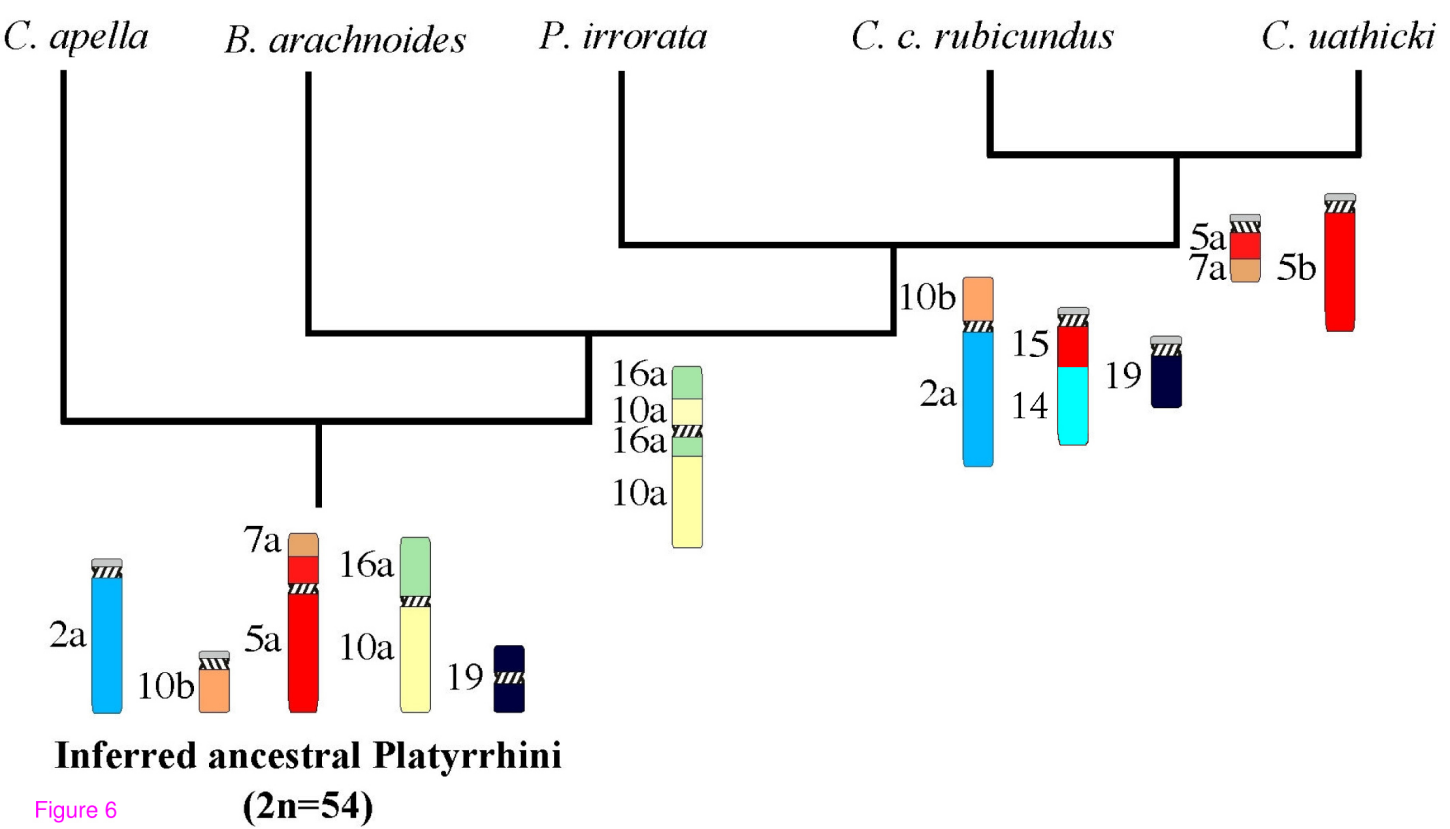
**Most parsimonious tree based on the binary chromosome character matrix (additional file **1). The analysis was made employing the maximum parsimony method using PAUP software.

These data were translated into a binary matrix (Additional file 1) and were then subjected to parsimony analysis. A single most parsimonious three was obtained (Figure 6) comprising 45 steps with a consistency index of 0.978, a retention index 0.909 and a homoplasy index of 0.022.

## Discussion

The G-, C-banding and NOR-staining results on *P. irrorata *and *C. c. rubicundus *obtained in this study are in agreement with previously published data [10], including the difference in the diploid number for males and females in *C. c. rubicundus *[10,15-17].

Our results by chromosome painting in *P. irrorata *and *C. c. rubicundus*, as well as those from *C. utahicki *[19], demonstrated that these species conserved all human homologous syntenic associations found in the inferred ancestral New World primate karyotype (3a/21, 5/7a, 2b/16b, 8a/18, 14/15a and 10a/16a) [27]. The morphology and banding pattern of the 3a/21 homologues in *C. c. rubicundus *and *C. utahicki *is similar to the ancestral Platyrrhini type, while in *P. irrorata *it was modified by a paracentric inversion. The biarmed chromosome form 5/7a found in *P. irrorata *is also similar to the ancestral Platyrrhini type, whereas in *C. c. rubicundus *and *C. utahicki *this association is found on a presumably derived acrocentric chromosome. *Cacajao *further shows human chromosome 5 homologues fissioned into 5a_1 _and 5a_2_. A similar fission was previously found in Atelinae [24], however, involving different break points. Therefore, these fissions represent no synapomorphy, but rather occurred independently in the two clades. Chromosome forms 2b/16b and 8a/18 each showed similar morphology compared to homologues from other Platyrrhini and are therefore considered ancestral traits. The association 14/15a was observed in an acrocentric chromosome in *P. irrorata*, while in *C. utahicki *it has fused with chromosome 20 forming the association 20/15/14 on chromosome 1. In *C. c. rubicundus *it was found fused with the human 3/20 homolog, leading to the association 3/20/15/14. Finally, a pericentric inversion was observed for chromosome form 10a/16a in all species analyzed here, resulting in chromosome form 16a/10a/16a/10a. This derived inversion has also been found previously in *Callicebus *[28], indicating that *Pithecia*, *Chiropotes *and *Cacajao *are sister groups of *Callicebus*. This observation is in agreement with recent classifications based on molecular data, supporting the classification of subfamilies Pitheciinae and Atelinae as sister groups included in the family Atelidae [8,12].

A comparative chromosome analysis of the three Pitheciinae species shows further synapomorphies shared between *C. c. rubicundus *and *C. utahicki *(fission of 5/7, resulting in separate 5a1 and 5a2 segments, and fusion 20/15/14), shared between *P. irrorata *and *C. c. rubicundus *(acrocentric 7b, acrocentric 12) and for Pitheciinae in general (fusion 2a/10b, acrocentric 15/14 and acrocentric 19). In contrast, no derived chromosome forms shared between *P. irrorata *and *C. utahicki *were found.

Our phylogenetic analysis suggests that *P. irrorata*, *C. c. rubicundus *and *C. utahicki *are a monophyletic group. Further, and as expected, the chromosomal data supported by exclusive synapomorphies demonstrates that *C. c. rubicundus *and *C. utahicki *are sister taxa. Moreover, *P. irrorata *was placed in a more basal position, having retained a karyotype closer to that of the inferred ancestral Platyrrhini. This phylogenetic arrangement supports previously published phylogenies [5-8,12], and also the phenetic inferences [10].

## Conclusions

In conclusion, this comparative chromosomal analysis clarifies some intrageneric relationships within Pitheciinae, while the position of this group in relation to *Callicebus *and *Aotus *remained undefined. Additional comparative high-resolution molecular cytogenetic studies will be necessary to precisely define the rearrangements between *Aotus *and *Callicebus *to clarify their phylogenetic relationships with Pitheciinae.

## Authors' contributions

LFMF carried out the chromosome painting in *P. irrorata*, organized the data and wrote most of the paper. PJSA carried out the conventional chromosome analysis of *P. irrorata *and performed the cladistic analysis. JCP participated of the techniques development and contributed to the discussion of data. EHCO carried out the conventional chromosome analysis of *C. c. rubicundus*. AP collected the samples, classified the species and discussed the phylogenetic implications of the data. MN and SM carried out the chromosome painting in *C. c. rubicundus *and discussed the phylogenetic implications of the data. CYN conceived of the study, and coordinated the study. All authors read and approved the final manuscript.

## Supplementary Material

Additional file 1 **Binary chromosome character matrix**. A table listing all the characters found in this study and heir alternative states. Click here for file
