# Supplementary material for: Chromosome phylogeny of the subfamily Pitheciinae (Platyrrhini, Primates) by classic cytogenetics and chromosome painting
Source: BMC Evol Biol. 2010 Jun 20;10:189. doi: 10.1186/1471-2148-10-189 (PMC2905426; doi:10.1186/1471-2148-10-189)
Supplement: Additional file 1 — Binary chromosome character matrix. A table listing all the characters found in this study and heir alternative states. [file 1471-2148-10-189-S1.DOC]

Additional data: Binary numeric matrix with the respective characters.

|  | CHARACTER | PIR | CCR | CUT | CAP | BAR |
| --- | --- | --- | --- | --- | --- | --- |
| 1 | 5/7a fission into 5a1 and 5a2 | 0 | 1 | 1 | 0 | 0 |
| 2 | 5a/5b/7a | 1 | 0 | 0 | 1 | 0 |
| 3 | 7b (acrocentric) | 1 | 1 | 0 | 1 | 1 |
| 4 | 10a/16a (parac. inv.) | 1 | 1 | 1 | 0 | 1 |
| 5 | 10b (acrocentric, not assoc.) | 0 | 0 | 0 | 1 | 1 |
| 6 | 2a/10b | 1 | 1 | 1 | 0 | 0 |
| 7 | 2a not assoc. | 0 | 0 | 0 | 1 | 1 |
| 8 | 15/14 (acrocentric) | 1 | 1 | 1 | 0 | 0 |
| 9 | 20/15/14 | 0 | 1 | 1 | 0 | 0 |
| 10 | 20 (acrocentric) | 1 | 1 | 1 | 0 | 1 |
| 11 | 13 (acrocentric, not assoc.) | 0 | 1 | 1 | 1 | 1 |
| 12 | 15b (acrocentric, not assoc.) | 0 | 1 | 1 | 1 | 1 |
| 13 | 22 (acrocentric, not assoc.) | 0 | 1 | 1 | 1 | 1 |
| 14 | 3a/21 (acrocentric) | 0 | 1 | 1 | 1 | 1 |
| 15 | 8b (acrocentric) | 1 | 1 | 1 | 0 | 0 |
| 16 | 8b (peric. inv.) | 0 | 0 | 0 | 1 | 1 |
| 17 | 12 (acrocentric) | 1 | 1 | 0 | 1 | 1 |
| 18 | 19 (acrocentric) | 1 | 1 | 1 | 0 | 0 |
| 19 | 19 (peric. inv.) | 0 | 0 | 0 | 1 | 1 |
